# A Mechanism for the Inhibition of DNA-PK-Mediated DNA Sensing by a Virus

**DOI:** 10.1371/journal.ppat.1003649

**Published:** 2013-10-03

**Authors:** Nicholas E. Peters, Brian J. Ferguson, Michela Mazzon, Aodhnait S. Fahy, Ewelina Krysztofinska, Raquel Arribas-Bosacoma, Laurence H. Pearl, Hongwei Ren, Geoffrey L. Smith

**Affiliations:** 1 Section of Virology, Department of Medicine, Imperial College London, Norfolk Place, London, United Kingdom; 2 Department of Pathology, Cambridge University, Cambridge, United Kingdom; 3 Cancer Research UK DNA Repair Enzymes Group, Genome Damage and Stability Centre, University of Sussex, Falmer, Brighton, United Kingdom; University of Alberta, Canada

## Abstract

The innate immune system is critical in the response to infection by pathogens and it is activated by pattern recognition receptors (PRRs) binding to pathogen associated molecular patterns (PAMPs). During viral infection, the direct recognition of the viral nucleic acids, such as the genomes of DNA viruses, is very important for activation of innate immunity. Recently, DNA-dependent protein kinase (DNA-PK), a heterotrimeric complex consisting of the Ku70/Ku80 heterodimer and the catalytic subunit DNA-PKcs was identified as a cytoplasmic PRR for DNA that is important for the innate immune response to intracellular DNA and DNA virus infection. Here we show that vaccinia virus (VACV) has evolved to inhibit this function of DNA-PK by expression of a highly conserved protein called C16, which was known to contribute to virulence but by an unknown mechanism. Data presented show that C16 binds directly to the Ku heterodimer and thereby inhibits the innate immune response to DNA in fibroblasts, characterised by the decreased production of cytokines and chemokines. Mechanistically, C16 acts by blocking DNA-PK binding to DNA, which correlates with reduced DNA-PK-dependent DNA sensing. The C-terminal region of C16 is sufficient for binding Ku and this activity is conserved in the variola virus (VARV) orthologue of C16. In contrast, deletion of 5 amino acids in this domain is enough to knockout this function from the attenuated vaccine strain modified vaccinia virus Ankara (MVA). *In vivo* a VACV mutant lacking C16 induced higher levels of cytokines and chemokines early after infection compared to control viruses, confirming the role of this virulence factor in attenuating the innate immune response. Overall this study describes the inhibition of DNA-PK-dependent DNA sensing by a poxvirus protein, adding to the evidence that DNA-PK is a critical component of innate immunity to DNA viruses.

## Author Summary

To mount an immune response to an invading bacterium or virus (pathogens), the host must detect foreign molecules from the pathogen. Pathogens have conserved features called pathogen associated molecular patterns (PAMPs) that are distinct from host cells and which are recognised by the host using specific sensors (called pattern recognition receptors, PRRs). One example of a PAMP is DNA in the cytoplasm. Cytoplasmic DNA activates the innate immune system, but the PRRs responsible remain incompletely understood. One such PRR, DNA-PK, was identified recently. Here we demonstrate that vaccinia virus (VACV), the vaccine used to eradicate smallpox, encodes a protein called C16 which binds to the DNA-PK complex and prevents it from sensing foreign DNA and activating the immune response. A VACV strain lacking C16 showed reduced virulence and, consistent with this, the host mounted a stronger innate immune response to infection. This illustrates the importance of DNA-PK as a sensor for foreign DNA, and increases understanding of the interaction between VACV and the host. It also illustrates how the study of virulence factors of pathogens can lead to the identification of novel components of the immune system.

## Introduction

The battle between host and pathogen has driven the evolution of the immune system and of pathogens. The result of this on-going fight is the development of sophisticated host detection and response systems and also of elegant pathogen subversion mechanisms [1], [2]. As part of the innate immune response, pattern recognition receptors (PRRs) detect an invading pathogen and induce the production of cytokines and chemokines [3], [4]. Not surprisingly evolution has produced PRRs that bind to conserved, essential molecules of pathogens (pathogen-associated molecular patterns, PAMPs), making it hard for the pathogen to escape detection. For example, lipopolysaccharide (LPS) is an essential component of the outer membrane of Gram-negative bacteria and is detected by toll-like receptor (TLR) 4 [5]. Similarly, during virus infection, intracellular viral nucleic acids are detected by our innate immune system [4]. Since it is difficult to alter their genomes to escape detection, viruses have evolved proteins that counteract host detection mechanisms by binding and inhibiting signalling molecules [2]. Vaccinia virus (VACV) is a prime example of this evolutionary strategy because it encodes in its large double stranded (ds) DNA genome numerous proteins that inhibit the host innate immune system. It encodes, for example, at least 10 proteins which can block activation of nuclear factor kappa B (NF-κB), for example proteins N1 [6], [7], A46 and A52 [8]–[10], B14 [11], [12], K7 [13], M2 [14], K1 [15], E3 [16], C4 [17], and A49 [18] and others that block activation of interferon regulatory factor (IRF)-3 such as A46 [10], K7 [13], C6 [19] and N2 [20]. In addition, protein B13 inhibits caspase 1 thereby blocking production of IL-1β downstream of AIM2-mediated detection of foreign DNA [21]. However, although VACV has a dsDNA genome that stimulates the innate immune system, there have been no descriptions of VACV proteins capable of directly inhibiting the detection of its DNA genome by PRRs. One reason for this is that, until recently, the PRRs that detect intracellular DNA of pathogens have been poorly understood.

Recently, DNA-dependent protein kinase (DNA-PK) was identified as a PRR for DNA and DNA viruses and shown to activate IRF3-dependent innate immunity [22]. DNA-PK is best known as a large DNA repair complex consisting of Ku70, Ku80 (which together form the Ku heterodimer) and the catalytic subunit DNA-PKcs. To promote DNA repair, Ku binds to free ends of DNA, which induces a conformational change leading to the recruitment of DNA-PKcs via the C-terminal domain of Ku80 [23]–[25]. However, in addition to its role in DNA repair, DNA-PK is a critical component of IRF3-mediated innate immune DNA sensing in murine embryonic fibroblasts (MEFs) and adult murine skin fibroblasts. This discovery added to a growing list of putative DNA sensors which have been identified following initial descriptions that DNA activates an IRF3-dependent pathway [26], [27] These include DAI [28], AIM2 [29]–[32], RNA-polymerase III [33], [34], LRRFIP1 [35], DHX9/DHX36 [36], IFI16 [37], DDX41 [38], MRE11 [39] and cGAS [40]. Furthermore, there is an additional molecule, barrier to autointegration factor (BAF), which has not been shown to activate IRF3 or other innate immune signalling pathways but nonetheless has a critical function as a cytoplasmic DNA-binding molecule that inhibits poxviral DNA replication, and this function of BAF is inhibited by the VACV B1 protein kinase [41]. It is only beginning to become clear, however, which sensors act in which cell types to detect which pathogens [42]. It is evident these DNA sensors have different patterns of cellular expression and have differential preferences for the type of DNA. For example, AIM2 responds preferentially to cytoplasmic DNA in macrophages by forming an inflammasome leading to IL-1β and IL-18 secretion or pyroptotic cell death. The importance of AIM2 in the response to poxviruses and cytoplasmic bacteria has been demonstrated by the observation that mice lacking AIM2 are more susceptible to these pathogens [43], [44]. AIM2 does not, however, stimulate IRF3 activation. RNA-polymerase III binds to, and transcribes, AT-rich DNA and produces a 5′ triphosphate RNA molecule which acts as a stimulatory ligand for RIG-I [33], [34], although which cell types and pathogens this is most important for have not yet been identified. LRRFIP1 and IFI16 have little specificity for the type of DNA detected and have been characterised mostly in macrophage cell lines [35], [37]. Conversely, DHX9/DHX36 and DHX41 function in plasmacytoid dendritic cells (pDCs) and myeloid dendritic cells respectively, and are stimulated principally by intracellular DNA bearing CpG motifs or by dsDNA respectively [36], [38]. cGAS was identified following the identification that cyclic guanosine monophosphate-adenosine monophosphate (cGAMP) bound to, and activated, the adaptor molecule STING [45]–[49]. However, of the DNA sensors that lead to IRF3-mediated IFN production, only DNA-PK has been shown to function *in vivo*. Mice lacking DNA-PK infected with MVA or herpes simplex virus (HSV) type 1 were deficient in the upregulation of pro-inflammatory cytokines and chemokines [22].

The interest in VACV derives historically from its use as the vaccine that eradicated smallpox, caused by the related orthopoxvirus, variola virus (VARV) [50]. After eradication was achieved interest in VACV continued due to its development as an expression vector [51], [52] that has application for development of new live vaccines [53]–[55]. More recently, VACV has been utilised as a tool for studying host-pathogen interactions that has shed light on immune system functions [56] and how VACV exploits cell biology for rapid dissemination [57], [58]. VACV strain modified virus Ankara (MVA) is a promising vaccine vector [59] and is highly attenuated due to extensive passage in chicken embryo fibroblasts leading to several large genome deletions [60]. These deletions removed several immunomodulatory genes [61], and smaller lesions in other genes have resulted in loss of protein function [62].

VACV strain Western Reserve (WR) protein C16 is an intracellular virulence factor and is conserved among orthopoxviruses, including VARV and MVA [63]. Mice infected with a virus lacking C16 (vΔC16) had more leukocytes infiltrating infected tissue, lost less weight and showed fewer signs of disease compared with both wild-type and revertant viruses. However, the mechanism of action of the C16 protein was not understood [63].

In this study the mechanism by which VACV protein C16 influences the immune response has been investigated and it is shown that, by binding to the Ku70/80 complex, C16 blocks DNA sensing in fibroblasts. The discovery of the interaction between C16 and Ku led to the decision to investigate the potential role of DNA-PK in innate immunity, leading to description of DNA-PK as a PRR for cytoplasmic DNA [22]. The interaction between C16 and Ku is via the C-terminal region of C16 that interacts directly with Ku70/80 and thereby reduces its ability to bind to DNA. This C-terminal region is highly conserved in both the VARV and MVA C16 orthologues; however, although VARV C16 can interact with Ku70/80, a small internal deletion in MVA C16 knocks out this binding activity. *In vivo* and compared to control viruses, vΔC16 caused greater induction of cytokines in the first 48 h of infection, consistent with C16 functioning as an inhibitor of the innate immune response. This study therefore highlights the *in vivo* importance of DNA-PK as a DNA sensor and describes how a DNA virus has evolved to inhibit DNA sensing as a way to subvert the detection of its genome by the host.

## Results

### C16 binds the Ku heterodimer

**Figure 1:**
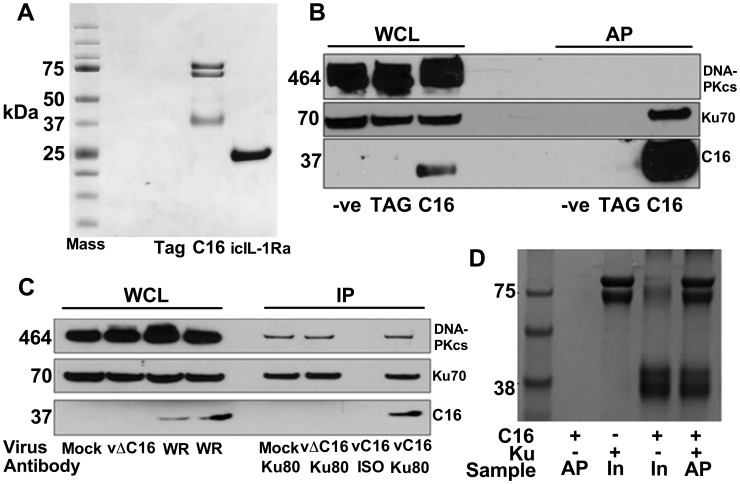
C16 interacts with the Ku heterodimer. **A**) Purification of TAP-tagged C16 by tandem affinity protein purification (TAP). C16-TAP was purified in parallel with the TAP-tag alone or with icIL-1Ra-TAP followed by resolution with NuPAGE (Invitrogen) and staining with Coomassie brilliant blue. **B**) Immunoblotting. Whole cell lysates (WCL) from HEK293Trex cells expressing TAP-tagged C16 or the same cell line not induced to express C16 (-ve) or a cell line expressing the TAP-tag alone (TAG) were analysed by SDS-PAGE and immunoblotting. Lysates from these cells were also affinity purified (AP) using streptavidin beads and analysed in parallel. Blots were probed with antibodies shown on the right. **C**) Immunoprecipitation. HeLa cells were infected with wild-type VACV (WR) or vΔC16, or mock infected and whole cell lysates (WCL) were analysed by SDS-PAGE and immunoblotting. Alternatively, cell extracts were immunoprecipitated with anti-Ku 80 antibody (Ku80) or an isotype control (ISO) 6 h post-infection and the immunoprecipitates were analysed in parallel. **D**) C16 interacts with Ku directly. Co-precipitation with recombinant Strep-tagged Ku70/Ku80ΔC pulling down recombinant C16. Ku70/Ku80ΔC was immobilized on Strep-Tactin beads and then incubated with C16 as described in Methods. The lanes represent beads with C16, Ku or both proteins and are either affinity purified (AP) or samples with 10% of input (In) samples. The positions of molecular size markers (kDa) are indicated. Each experiment was repeated three times (A–C) and twice (D).

To investigate how C16 modulates the host immune response, C16 was tandem affinity purification (TAP)-tagged [64], expressed inducibly in HEK293 TRex cells and the C16 protein complexes were purified and analysed by SDS-PAGE and mass spectrometry. C16 was purified using this method in parallel with a control protein, the intracellular IL-1 receptor antagonist (icIL-1Ra) that has a similar motif to C16 at the C-terminus [63], [65]. C16 co-purified with two proteins of 70 and 80 kDa that were identified by liquid chromatography mass spectrometry (LC/MS) as the two components of the Ku heterodimer, Ku70 and Ku80. These proteins did not co-purify with icIL-1ra-TAP and no proteins were detected from the non-induced C16 cell line or a cell line expressing the TAP-tag alone (Figure 1A).

Confirmation of this interaction was carried out in several ways. Firstly, C16 complexes were affinity purified from the HEK293 cell line and immunoblotted for Ku70 and DNA-PKcs showing that C16 binds to the Ku complex but not the third DNA-PK component, DNA-PKcs (Figure 1B). Secondly, immunoprecipitation of endogenous Ku80 from cells infected with VACV WR, or vΔC16 as a control, confirmed that this interaction was observed during virus infection when both proteins were expressed at endogenous levels (Figure 1C). Finally, to test whether the binding of C16 to Ku was direct, recombinant C16 protein was expressed and purified from *E. coli* and was then incubated with Strep-tagged Ku70/Ku80ΔC that had been purified from insect cells infected with recombinant baculoviruses. The Strep-tagged Ku70/Ku80ΔC complex was then re-purified on a Strep-Tactin matrix and analysed by SDS-PAGE. This showed that C16 co-purified with the Ku proteins (Figure 1D) and thereby confirmed that the interaction between Ku70/80 and C16 was direct. The Ku70/Ku80ΔC complex lacks the small C-terminal domain implicated in binding DNA-PKcs, suggesting that C16 does not bind Ku in direct competition with the catalytic subunit of DNA-PK. Collectively, these experiments showed that C16 interacts directly with the Ku complex, both in cells and *in vitro*, and can be observed with endogenous proteins in the context of VACV infection.

### The C-terminal region of C16 mediates the interaction with Ku70/80

**Figure 2:**
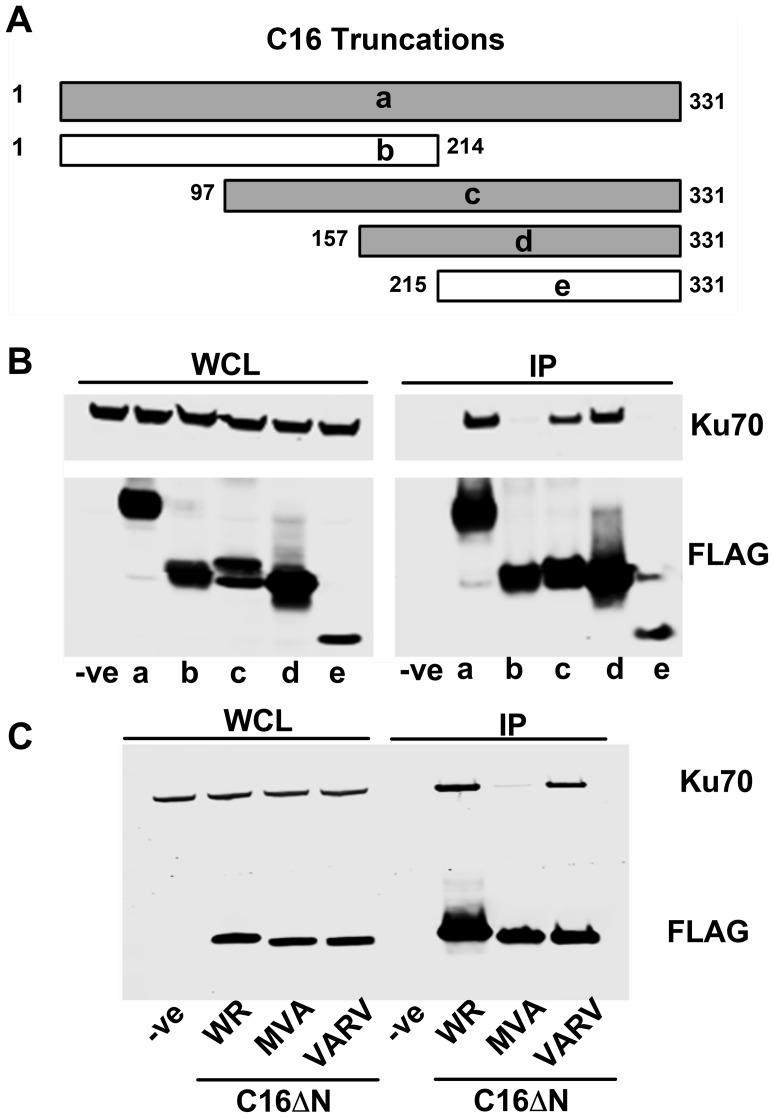
The C-terminal domain of C16 is sufficient for binding to Ku. A) Schematic of C16 truncations used to map Ku-interacting domain. Grey bars represent C16 fragments able to bind Ku70 and white bars represent weak or no binding. **B**) FLAG-tagged full length C16 (1–331); a, or C16 fragments representing amino acid residues 1–214 (b), 97–331 (c), 157–331 (d) or 215–331 (e) were expressed by transfection in HEK293 cells and cell lysates were analysed by SDS-PAGE and immunoblotting. In parallel, extracts form these cells were affinity purified with Strep Tactin beads and the affinity purified proteins were immunoblotted with anti-Ku70 or anti-FLAG Abs. **C**) TAP-tagged C16 from VACV WR (WR C16ΔN), VACV MVA (MVA C16ΔN) and VARV (VARV C16ΔN) corresponding to amino acid residues 157–331 of VACV WR C16 were expressed by transfection in HEK293 cells and then affinity purified Strep Tactin beads and then immunoblotted with anti-FLAG or anti-Ku70 Abs. WCL, whole cell lysate; IP, immune precipitated. The experiments shown in B and C were done 3 times and twice, respectively.

To determine which region of C16 is needed to bind Ku, FLAG-tagged fragments of C16 (Figure 2A) were expressed in HEK293T cells and tested for binding to the Ku complex (Figure 2B). Full-length C16 (amino acid residues 1–331) co-precipitated with Ku70/80, as did the C16 fragments containing amino acid residues 97–331 and 157–331. In contrast, the N-terminal region comprising amino acid residues 1–214, as well as the C-terminal amino acids 215–331, did not co-precipitate with Ku. These data show that the C-terminal residues 157–331 of C16 are sufficient for binding Ku (Figure 2B).

Whilst VACV C16 is highly conserved among VACV strains, there are minor differences in the orthologues of C16 encoded by VARV and VACV strain MVA. In MVA the C16 orthologue has an internal deletion of 5 amino acids (residues 277–281) (Figure S1) but was detected at levels similar to VACV WR C16 during infection [63]. In comparison, the VARV-GBR46 differs by 6 (1.8%) amino acids spread across the protein. To test if these changes affect the binding of C16 to the Ku complex, alleles of MVA and VARV C16 corresponding to VACV WR residues 157–331 were expressed in HEK293T cells and then assessed for binding to Ku70 (Figure 2C). In this assay, the VARV C16 orthologue interacted with Ku70 but the MVA orthologue showed severely diminished binding. These data suggest not only that amino acids 277–281 are important for the interaction between C16 and Ku, but also that whilst VARV protein C16 targets Ku70, that ability has been lost in the attenuated MVA strain of VACV.

### C16 disrupts the binding of Ku70/80 to DNA

**Figure 3:**
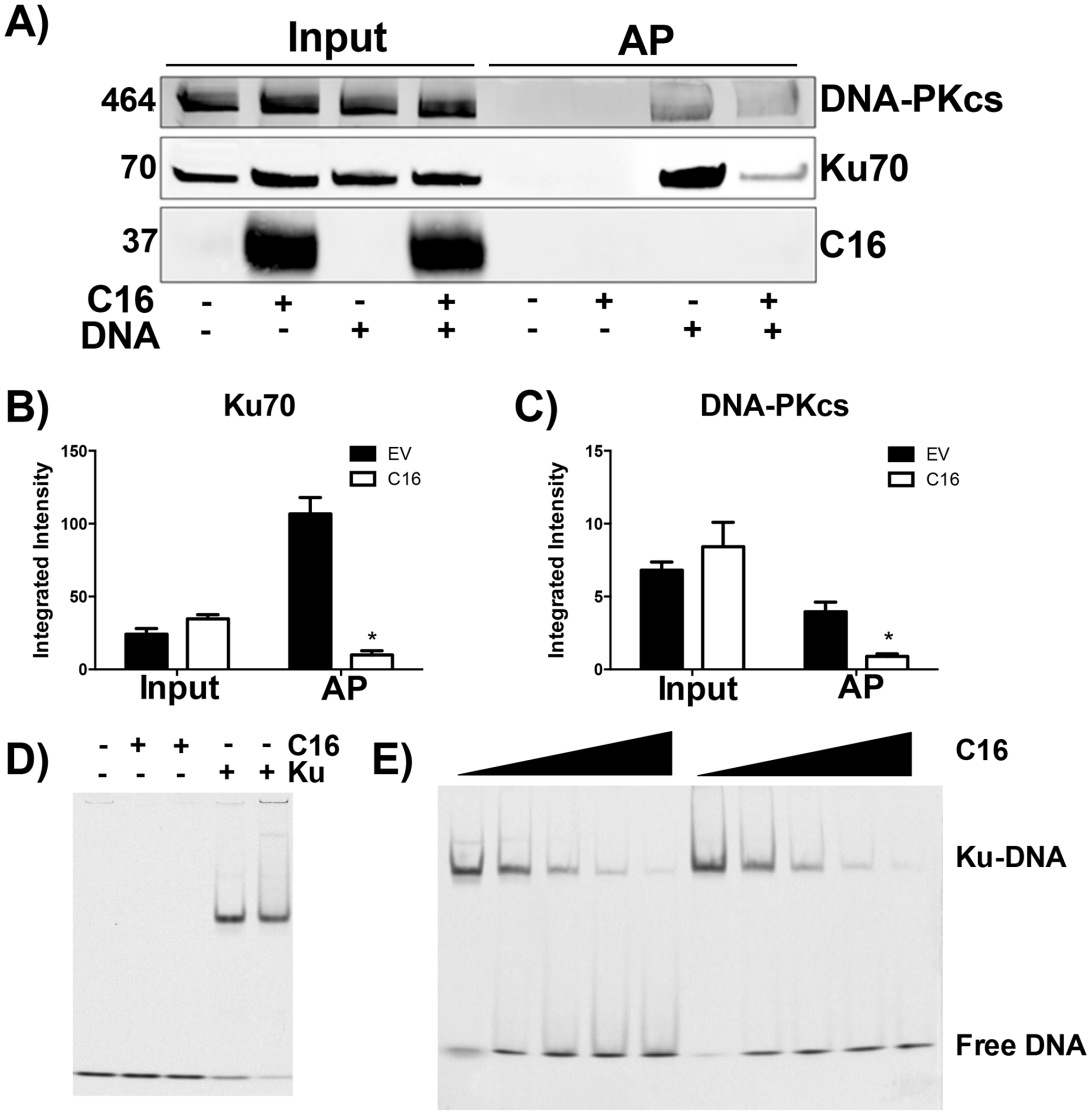
C16 affects binding of DNA-PK to DNA. (A) HeLa cells were transfected with 2 µg/ml pcDNA4/TO (EV) or pcDNA4/TO-coC16 (C16) for 24 h followed by 2 µg/ml biotinylated ISD DNA for 1 h. Biotinylated DNA was purified from cytoplasmic cell lysates with streptavidin beads and associated proteins were resolved by SDS-PAGE and immunoblotted with C16, Ku70 and DNA-PKcs antibodies. Integrated intensity of (**B**) Ku70 and (**C**) DNA-PKcs was calculated using infrared imaging by a Licor Odyssey scanner from three independent experiments. * p<0.05. (**D, E**). Electrophoretic mobility shift assay showing that the C-terminal domain of C16 inhibits binding of Ku to DNA. (**D**) A Cy3-labelled 19-bp duplex oligonucleotide (10 nM) was incubated with (+) Ku70/Ku80ΔC (20 nM) or recombinant C16 [aa 157–331] (20 nM). (**E**) A Cy3-labelled 19-bp duplex oligonucleotide (10 nM) was incubated with Ku70/Ku80ΔC (20 nM) in the presence of increasing amounts (20, 40, 80, 120 and 160 nM) of recombinant C16 [aa 157–331]. Duplicate samples were analysed on a 5% polyacrylamide gel. The positions of free DNA and DNA-Ku complexes are shown.

The consequence of C16 binding to Ku was analysed by testing the ability of C16 to interrupt the interaction between DNA and the Ku70/80 heterodimer. Biotinylated DNA was transfected into cells and DNA:protein complexes were isolated from the cytoplasm via the biotin tag. In whole cell lysates it was noted that the level of DNA-PKcs and Ku70 were similar in the presence or absence of transfected DNA, and whether or not C16 was expressed (Figure 3A–C). In cells expressing C16, however, the amounts of both Ku and DNA-PKcs that co-purified with DNA were reduced substantially compared with cells transfected with empty vector. This was not due to degradation of DNA-PK components because measurement of the levels of Ku70 and DNA-PKcs showed a slight, but statistically insignificant, increase in the expression level of DNA-PK components in the presence of C16. These observation indicated that C16 inhibits the interaction of DNA-PK with DNA (Figure 3A). Quantification of this result by immunoblotting from triplicate experiments confirmed a statistically significant reduction in DNA-PK components binding to DNA in the presence of C16 (Figure 3B, C). It was also noted that C16 did not co-purify with DNA implying that C16 does not bind to DNA directly.

To investigate if C16 was sufficient to block Ku binding to DNA, purified Ku70/Ku80 was incubated with DNA in the absence or presence of increasing concentrations of the purified C-terminal domain of C16 and DNA protein complexes were analysed by electrophoretic mobility shift assay (EMSA) (Figure 3D, E). This showed that the C-terminal domain of C16, inhibited the electrophoretic shift induced by Ku. Furthermore, C16 alone did not induce an electrophoretic shift (Figure 3D), supporting the observation that C16 does not bind to biotinylated DNA (Figure 3A). At a high molar C16∶Ku ratio of approximately 6∶1, Ku was no longer able to shift DNA. Together, these data demonstrate that the C16 C-terminal domain is sufficient for inhibiting the interaction between Ku and DNA.

Overall, these data show that C16 functions to inhibit the binding of Ku70/80 to DNA, thereby greatly reducing the interaction of the DNA-PK complex with foreign DNA in the cytoplasm. This proposed model is illustrated in Figure S2.

### C16 inhibits DNA-PK-mediated DNA sensing

**Figure 4:**
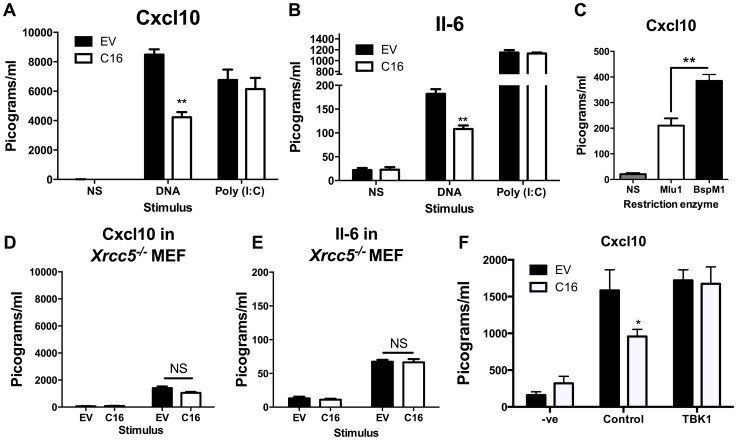
C16 inhibits DNA sensing, but not RNA sensing. (**A, B**) MEFs were mock transfected (NS), transfected with 200 ng/ml pcDNA4/TO (EV) or pcDNA4/TO encoding codon optimised C16 (coC16) and 5 µg/ml concatamerised DNA or 2 µg/ml poly (I∶C). The presence of Cxcl10 or Il-6 was measured in the supernatants at 24 h post transfection by ELISA. (**C**) MEFs were mock transfected (NS), or transfected with 2 µg/ml of purified pcDNA4/TO-coC16 linearised previously with the indicated restriction endonuclease enzyme. Cxcl10 or Il-6 was measured in the supernatants as in A,B. (**D, E**) *Trp53^−/−^Xrcc5^−/−^* (Ku80 null) MEFs were transfected with 200 ng/ml pcDNA4/TO (EV) or pcDNA4/TO encoding codon optimised C16 (C16) and 5 µg/ml concatamerised DNA. The presence of (**D**) Cxcl10 and (**E**) Il-6 were measured by ELISA in the supernatant at 24 h post transfection. (**F**) MEFs were mock transfected (-ve), transfected with 200 ng/ml pcDNA4/TO or pcDNA4/TO encoding codon optimised C16 (coC16) and 5 µg/ml concatamerised DNA along with either control vector or vector expressing TBK1. Error bars +/− SEM (n = 5), ** p<0.01, NS = non-significant. Each experiment was repeated a minimum of three times.

Since C16 interacted with Ku70/80 and inhibited its binding to DNA, we proposed that VACV C16 had evolved to inhibit Ku-mediated DNA sensing. C16 was therefore assessed for its ability to block the production of cytokines by MEFs in response to cytoplasmic DNA stimulation. Due to the difficulty of expressing C16 without the transfected DNA plasmid itself stimulating innate immune signalling, a plasmid expressing C16 was co-transfected simultaneously with larger molar quantities of linear immunomodulatory DNA and the amount of Cxcl10 and Il-6 produced were measured by ELISA 24 h later. Under the conditions tested, co-transfection of C16, compared with a control plasmid, reduced the production of Cxcl10 and Il-6 in response to DNA by approximately 50 per cent, but not poly I∶C (a dsRNA mimic) (Figure 4A, B). This indicated that C16 inhibited the innate immune activation of these cells by DNA but not RNA, consistent with the described function of Ku70/80 in DNA sensing [22]. This inhibition was investigated further by digesting the plasmid encoding the C16 ORF with the restriction enzymes *Bsp*MI, which disrupted the C16 ORF, or *Mlu*I that cut the plasmid without affecting the C16 ORF. These linearised plasmids were then transfected into MEFs such that the dsDNA stimulus was also responsible for expression of the gene of interest, rather like the situation during DNA virus infection. When the C16 ORF remained intact (*Mlu*I digestion) the level of Cxcl10 induced was lower than when it was disrupted (*Bsp*MI digestion) (Figure 4C), supporting the hypothesis that C16 inhibits the innate immune response to DNA by MEFs.

MEFs lacking the Ku heterodimer induce lower levels of cytokines and chemokines induced upon stimulation with DNA [22]. However, the abrogation is not complete and there is residual signalling. This is explained (at least in part) by DNA-PKcs having DNA-binding capability independent of Ku [66], and also by the existence of other DNA sensing mechanisms. If C16 inhibited the production of Cxcl10 and Il-6 via its interaction with Ku, following DNA stimulation C16 might be expected to not influence the induction of these molecules in MEFs lacking Ku80, gene *Xrcc5*, and therefore lacking the Ku heterodimer [67]. This was tested and shown to be correct, although the overall level of cytokine induction was reduced as expected (Figure 4D, E). This suggests that the inhibition of DNA sensing mediated by C16 was dependent on its interaction with the Ku heterodimer. Together, these data suggest that C16 inhibited DNA sensing, but not RNA sensing, and that this was mediated by its interaction with Ku.

The hypothesis that C16 was disrupting DNA-sensing at the sensor level was tested further by overexpression of a molecule downstream in the signalling pathway. It was observed that, whilst C16 blocked DNA sensing when co-transfected with empty vector, this inhibition was overcome when TBK-1 was co-transfected with C16 (Figure 4F). This shows that the inhibitory effect exerted by C16 is upstream of this component in the DNA-sensing pathway. The effect on IRF3 translocation was also studied. MEFs were infected with wild-type virus (vC16) or a C16 deletion virus (vΔC16) and the location of IRF3 was examined by immunofluorescence. This experiment showed that infection of MEFs with these WR-based viruses did not induce IRF3 translocation (Figure S3), in contrast to the ability of MVA to activate this innate immune signalling pathway [22]. This is likely to be explained by a number of VACV proteins which have evolved to inhibit IRF3-mediated signalling, independent of C16 [13], [19], [20].

### Viruses lacking C16 induce greater production of cytokines *in vivo*

**Figure 5:**
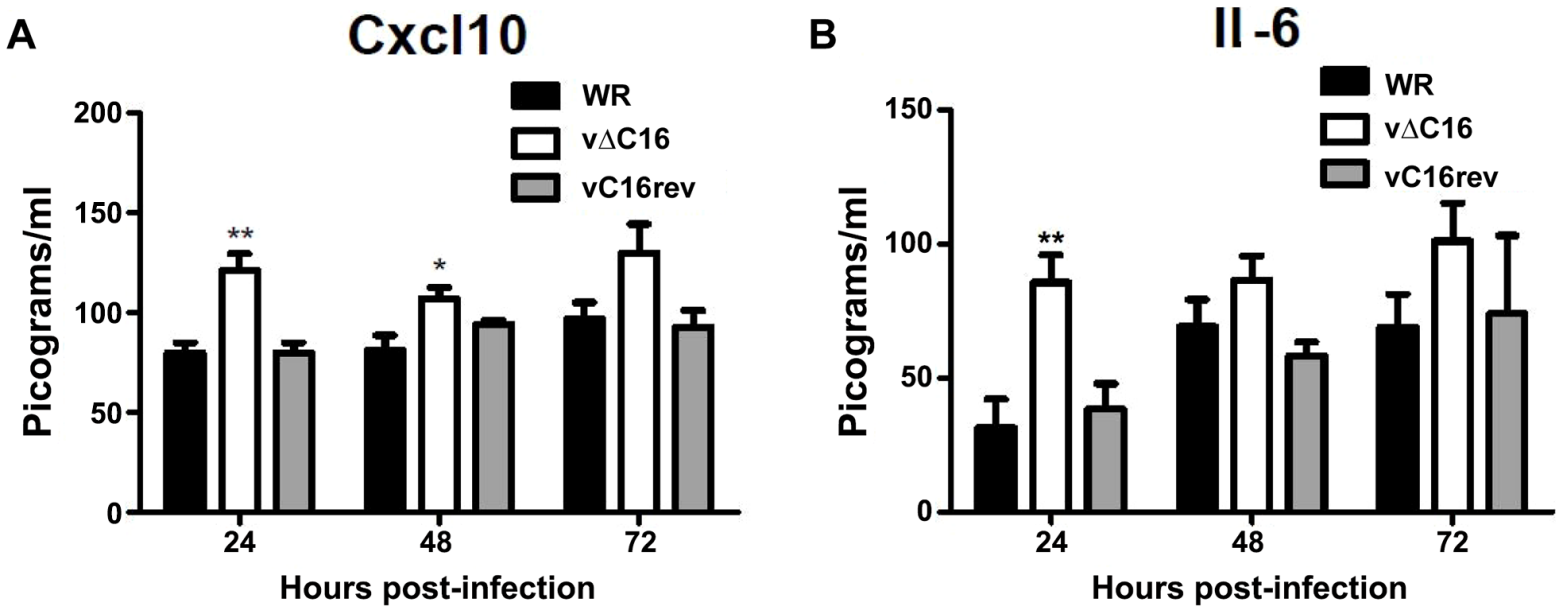
C16 affects Cxcl10 and Il-6 production in vivo. (**A, B**) Groups of five BALB/c mice were infected intranasally with 5×10^4^ pfu per mouse of wild-type (WR), C16 knockout (vΔC16) or revertant viruses. Mice were sacrificed at the indicated time points and the amount of (**A**) Cxcl10 and (**B**) Il-6 in BAL fluid was measured by ELISA. Error bars +/− SEM (n = 3). * p<0.05, ** p<0.01.

To assess the contribution of C16 to the innate immune response *in vivo*, mice were infected intranasally with either a plaque purified wild type VACV WR , vΔC16, or a revertant virus in which the *C16L* gene had been re-inserted into its original locus (Figure 5) [63]. VACV infection induced the production of Cxcl10 and Il-6 into the bronchoalveolar lavage (BAL) fluid, however, infection with VACV vΔC16 lead to an enhanced production of these cytokines. Consistent with the function of C16 inhibiting the innate immune response to DNA, this effect was significant in the first 2 d post infection (p.i.) at 24 and 48 h p.i. for Cxcl10, and at 24 h p.i. for Il-6. C16 is a virulence factor, causing increased weight loss and reducing the number of leukocytes recruited to the lungs of mice infected with VACV [63]. Overall, data presented in this study, demonstrate that DNA-PK-mediated activation of the innate immune response to VACV is of biological significance *in vivo* and that C16 is capable of inhibiting this function.

## Discussion

The interactions between virus and host proteins have led to discoveries about the function of multiple cellular systems, including the innate immune system. VACV encodes many inhibitors of both extracellular molecules and intracellular signalling cascades to help dampen down immune responses. Examples of extracellular immunomodulators include protein A41 that inhibits binding of chemokines to glycosaminoglycans, thereby preventing establishment of the concentration gradient of these molecules [68], [69]. Other examples include VACV proteins B18 and B8 as decoy receptors for type I and type II IFNs, respectively [70]–[74]. Examples of intracellular inhibitors of the innate immune system including the steroid biosynthetic enzyme 3β-hydroxysteroid dehydrogenase that reduces the VACV-specific CD8+ T cell response to infection [75], [76], B14 that binds to IKKβ and thereby inhibits activation of NF-κB [11], C6 that inhibits activation of IRF3 by interacting with adaptor proteins involved in the activation of TBK1 [19] and which suppresses the immune responses to infection [77], [78]. Therefore, VACV is a useful tool for studying the innate immune system both to study known signalling mechanisms and discover novel molecules.

Here we define a viral inhibitor of DNA-PK, a protein complex described recently as a PRR that senses foreign DNA, including the genomes of DNA viruses [22]. Using an unbiased mass spectrometry approach, VACV protein C16 is shown to bind to the Ku70/80 heterodimer, part of the DNA-PK complex. This direct protein/protein interaction occurs in cells, during ectopic expression of C16 and during VACV infection, as well as *in vitro* using recombinant purified proteins. By binding Ku70/80, C16 is able to inhibit DNA, but not RNA, sensing in fibroblasts. Mechanistically, C16 achieves this inhibition by preventing the binding of Ku to DNA. This interaction has been localised to the C-terminal domain of C16, and is also independent of the C-terminal domain of Ku80. Interestingly, whilst C16 does not bind DNA-PKcs directly, and does not appear to bind Ku70/80 in direct competition with DNA-PKcs, it reduces the amount of DNA-PKcs bound to DNA. These observations are consistent with previous findings that, although DNA-PKcs can bind DNA directly, this interaction is greatly enhanced by the presence of the Ku heterodimer [66], [79], and that Ku70/80 is important for DNA sensing by the DNA-PK complex [22].

C16 was demonstrated to inhibit DNA-mediated activation of the innate immune system. Under the conditions tested this resulted in approximately a 50% reduction in the production of the pro-inflammatory molecules Cxcl10 and Il-6. The remaining DNA sensing capability is likely explained by both incomplete penetrance of MEFs with C16-encoding plasmid, the abundance of the DNA-PK and further Ku-independent DNA sensors, such as IFI16 and cGAS shown previously to be operational in MEFs [37], [46].

C16 has therefore evolved as a viral countermeasure to the detection of the VACV genome by the host innate immune system, and, as such, the loss of C16 contributes to the attenuation of this virus *in vivo*. The influence of C16 on virus virulence and the immune response to VACV infection in murine models has been described previously [63] and data in the present study add to the findings of that report. Infection of mice with VACV vΔC16 caused enhanced production of the chemokine Cxcl10 and the cytokine Il-6 in the lungs. Given the chemoattractive properties of these molecules, this likely explains the increased numbers of infiltrating leukocytes during infection with vΔC16 observed previously [63].

As with other viruses, the detection of the VACV genome is important for the host response to infection [22]. The discovery of a VACV protein that inhibits this process re-enforces this fact and shows the relevance of DNA-PK *in vivo* as a sensor of poxvirus DNA. In addition, the observation that VARV C16 can also bind to Ku70/80 is a strong indication that the pathogen that caused smallpox also evolved to inhibit DNA-PK-dependent DNA sensing. In contrast, the C16 orthologue encoded by VACV strain MVA has an internal deletion of five amino acids from its C16 orthologue that results in loss of binding to Ku70. Similarly, internal deletion of 6 amino acids from MVA protein 183, the orthologue of VACV WR protein B14, ablated its ability to inhibit NF-κB activation [62]. The failure of MVA to inhibit detection of its genome may be partly responsible for strong innate and adaptive immune response to this virus.

The role of DNA sensing in disease is an emerging field and its relevance to pathological processes beyond viral infection is beginning to be explored. Conditions such as Aicardi-Goutières syndrome [80] and systemic lupus erythematosus [29], [81], [82] have shown association with DNA sensing mechanisms, such as AIM2, and it is possible that DNA-PK, or other DNA sensors, contributes to the disease process. In the future it may be possible to exploit the interaction between C16 and Ku70/80 as a model for the development of small-molecule inhibitors to alleviate pathological processes caused by the accumulation of intracellular DNA.

In summary, this paper demonstrates an interaction between a known viral virulence factor and the Ku complex. This discovery led to the decision to investigate the potential role of DNA-PK in innate immunity, and consequently to the demonstration that it was a cytoplasmic DNA sensor that activates IRF3-dependent innate immunity [22]. To our knowledge the inhibition of DNA sensing by C16 represents the first viral interference with a dsDNA sensor shown to have an *in vivo* effect and adds weight to the hypothesis that DNA-PK is an important component of innate immunity. This work also strengthens the case for investigating the roles of microbial virulence factors due to the potential to discover novel features of the immune system.

## Materials & Methods

### Ethics statement

This work was carried out in accordance with regulations of The Animals (Scientific Procedures) Act (United Kingdom) 1986. All procedures were approved by the UK Home Office and carried out under the Home Office project licence PPL 70/7116.

### Mice

Groups of five female BALB/c mice between six and eight weeks old were anaesthetised and inoculate intranasally with 5×10^4^ plaque-forming units (PFU) of VACV strain WR intracellular mature virus (IMV) that had been purified by sucrose density gradient centrifugation and was diluted in 20 µl PBS. Mice were sacrificed at the specified time points under terminal anaesthesia with isofluorane and were exsanguinated from the subclavian artery. Bronchoalveolar lavage (BAL) fluid was harvested using five 200 µl lavages of lungs via the trachea and centrifuged to remove cellular debris.

### Plasmids

The C16 ORF was cloned into the pcDNA4 T/O expression plasmid using *Bam*H1 and *Not*1 sites. The TAP-tag sequence comprised one STREP (WSHPQFEK) and two FLAG (DYKDDDDK) sequences at the C terminus of the protein. C16 was codon optimised for expression in mammalian cells by GenScript (New Jersey, USA).

### Cell culture and transfection

HEK 293T and 293TRex (Life Technologies) cells were maintained in DMEM containing 10% FBS 100 U/ml penicillin and 100 µg/ml streptomycin. The 293Trex cell lines inducibly expressing C16, icIL-1ra and the TAP-tag alone were clonally selected using 5 µg/ml blasticidin and 100 µg/ml zeocin. MEFs were maintained in DMEM containing 15% FBS. HeLa cells were maintained in RPMI containing 10% FBS and 2 mM L-glutamine. Transfections were carried out with Lipofectamine 2000 (Life Technologies).

### Tandem affinity protein purification and mass spectrometry

Tandem affinity protein purification (TAP) was performed using Strep-Tactin superflow beads (IBA) and FLAG M2 agarose beads (Sigma-Aldrich) as described elsewhere [64]. Coomassie stained bands from tandem affinity purification procedures were excised using a sterile scalpel and placed in 100 µl H_2_O (Sigma-Aldrich). Samples were analysed by liquid chromatography mass spectrometry (LCMS/MS) at the Centre for Systems Biology at Imperial College London.

### Immunoblotting

Cell lysates were separated by electrophoresis and transferred onto Immobilon P membranes (GE Heathcare). These membranes were blocked in 5% nonfat milk in TBS containing 0.1% Tween 20 for 1 h at room temperature. Membranes were probed with Abs against Ku70 (AbCam), Ku80 (Santa Cruz), DNA-PKcs (Upstate) or C16 (Generated by Harlan Sera-Lab). Ku80, or control Abs were used for immunoprecipitation from HeLa cell lysates. Chemiluminescence imaging was used to develop immunoblots and quantification was performed using a Licor Odyssey scanner and band intensity was analysed using Odyssey software (Licor Biotechnology).

### Immunofluorescence

Cells were seeded onto 15-mm glass coverslips, and subsequently infected with VACV WR at 5 pfu/cell for 3 or 6 h as indicated. Samples were fixed with 4% paraformaldehyde and permeabilised with PBS containing 0.2% Triton X-100 and blocked with 5% non-fat milk in PBS with 0.1% Tween for 1 h at 20°C. Incubation with anti-murine IRF3 (Life Technologies, Grand Island, NY) diluted in PBS with 1% non-fat milk for 1 h was followed by detection with alexa-fluor-conjugated secondary antibody (Life Technologies, Grand Island, NY). Cells were counterstained with DAPI and mounted with Mowiol. Images were obtained using a Zeiss Pascal 510 microscope and processed with Zeiss LSM software (Zeiss, Oberkochen, Germany)

### Enzyme-linked immunosorbent assay (ELISA)

Levels of Cxcl10 and Il-6 in cell supernatants or BAL fluid were measured using ELISA kits (R&D systems) according to the manufacturer's instructions.

### DNA pull down assay

Double stranded oligonucleotide DNA (sense sequence, TACAGATCTACTAGTGATCTATGACTGATCTGTACATGATCTACA) was biotinylated at its 3′ end and transfected into HEK293T cells using PEI (Sigma). After 30 min, cells were lysed with a buffer containing 100 mM Tris-Cl, pH 8, 0.2% Triton X-100, 2 mM MgCl_2_, 1 mM EDTA, and centrifuged at 600 *g* in a microcentrifuge and the pellet was discarded. Crude cytoplasmic extracts were obtained by a further centrifugation at 20,000 *g*. Biotinylated DNA was then purified from the supernatant using Streptavidin beads.

### Expression of purification of C16 from *E. coli*

BL21(DE3)-R3-pRARE2 cells expressing C-terminal His-tagged C16 (C16-His) from pET28a (Novagen) were lysed in lysis buffer (50 mM HEPES pH 7.4, 500 mM NaCl, 5% glycerol, 30 mM imidazole pH 7.5, 0.5 mM TCEP, 1 tablet of complete protease inhibitor (Roche), 0.1% Triton X-100) and cell debris was removed by centrifugation. His-C16 was affinity purified through a 1 ml His-TRAP column (GE Healthcare) using a step-wise gradient of imidazole as indicated. The eluted fractions were concentrated using a 10-kDa cut-off concentrator filter (Amicon) and further purified by size exclusion chromatography (Superdex 200 16/60, GE Healthcare).

### Expression and purification of Ku70 and Ku80 from baculovirus-infected insect cells

To generate pFBDM-Strep-Ku70/Ku80ΔC for insect cell expression of Ku heterodimer containing a C-terminally truncated Ku80ΔC (lacking residues 591–732), human cDNA sequence encoding Ku80^1–590^ was cloned into the *Xho*I and *Nhe*I sites of pFBDM, downstream of the p10 promoter. cDNA sequence encoding full length human Ku70 was sub-cloned into the *Bam*HI/*Not*I sites of the same plasmid, downstream of the polyhedron promoter and in frame with an N-terminal Strep tag II. Ku70/Ku80ΔC was expressed and purified as described [83], changing the first affinity step to Strep-Tactin resin (Qiagen).

### Electrophoretic mobility shift assays (EMSA)

A 5′ Cy3-labelled oligonucleotide (5′ GAAAGCTATGGGCGCGGTT 3′) was annealed to its complementary oligonucleotide to generate a 19-bp blunt-ended duplex substrate. Recombinant Ku70/Ku80ΔC and/or C16 (aa157–331) were incubated at the concentrations indicated in 10 ml binding buffer (20 mM Tris–HCl pH 7.5, 50 mM NaCl, 0.5% glycerol, 0.1 mg/ml BSA) containing 10 nM Cy3-labelled DNA substrate for 15 min at room temperature. After adding glycerol to 5% total volume, the protein∶dsDNA mixtures were fractionated on a 5% native PAGE gel (37∶1 acrylamide∶bis-acrylamide) run in 0.4× TBE at room temperature and 10 mA. The gel was subsequently scanned on a Fujifilm FLA-500 instrument using a 532-nm laser and Cy3 filter.

### Analysis of Ku complex – C16 mixtures

For the coprecipitation experiments, 300 µl of 26 µM purified Strep-tagged Ku70/Ku80ΔC protein was added to 200 µl Strep-Tactin Superflow Plus Beads (Qiagen) pre-equilibrated in PBS and was incubated on a roller for 1 h at 4°C. Beads were washed four times with buffer A (PBS supplemented with 0.2% BSA, 5 mM DTT, and 0.1% NP-40). Purified C16 in PBS was added (250 µl of 12 µM) and incubated with the beads for 7 h at 4°C on a roller. Beads were then washed four times with buffer A and resuspended in 4× SDS loading buffer (Invitrogen). As a control, purified C16 was also incubated with Strep-Tactin Superflow Plus Beads that had not been incubated previously with Strep-tagged Ku70/Ku80ΔC. The control sample was treated as described before. All samples were analysed by SDS-PAGE using 4%–12% NuPAGE Bis-Tris gels (Invitrogen) run in 1× MES buffer (Invitrogen).

### Statistical analysis

Statistical analysis was carried out using student's t-test with Welch's correction where necessary.

## Supporting Information

Figure S1 **Alignment of C16 from VACV WR, VACV MVA and VARV.** Primary amino acid sequences from VACV WR C16 (W) protein and its orthologues in VACV MVA (M) and VARV GBR46 (V) were aligned using ClustalW software. The underscored region (amino acids 157–331) represents the C-terminal portion used in Figure 2C.
(TIF)

Figure S2 **Proposed model of C16 action.** C16 inhibits the binding of Ku to DNA thereby preventing the assembly of DNA-PK on VACV DNA and subsequent signal transduction via IRF3.
(TIF)

Figure S3 **WR infection does not lead to IRF-3 activation.** MEFs were infected with either wild-type WR VACV (vC16) or a recombinant virus lacking C16 (vΔC16) for 3 or 6 hours as indicated and stained for IRF-3. Scale bar; 10 µm.
(TIF)
