# Supplementary figures and images for: A Mechanism for the Inhibition of DNA-PK-Mediated DNA Sensing by a Virus
Source: PLoS Pathog. 2013 Oct 3;9(10):e1003649. doi: 10.1371/journal.ppat.1003649 (PMC3789764; doi:10.1371/journal.ppat.1003649)

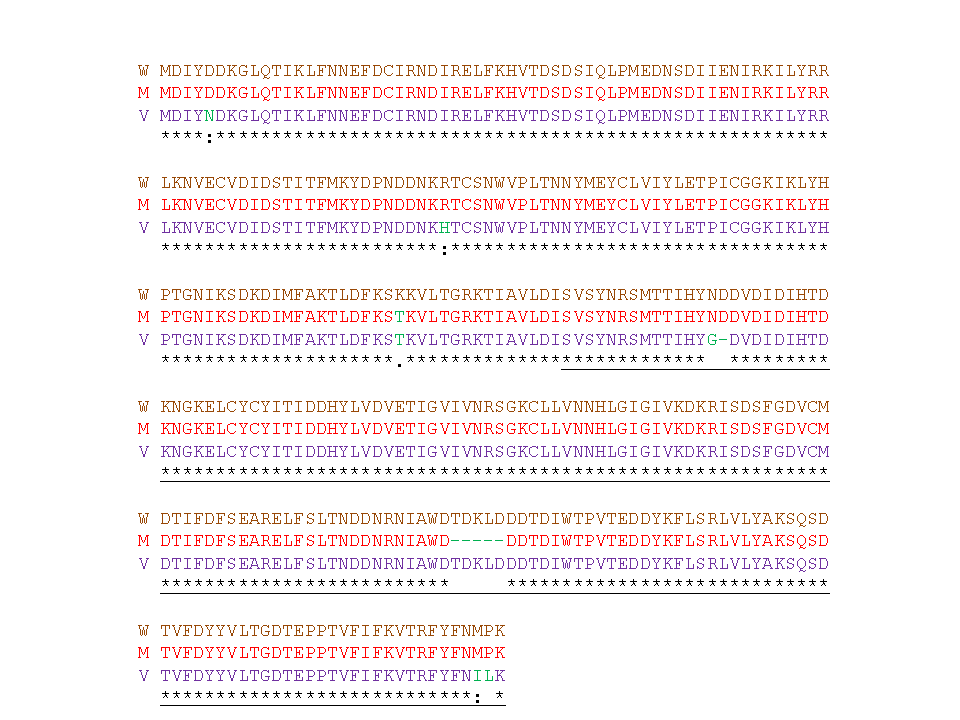

Supplement: Figure S1 — Alignment of C16 from VACV WR, VACV MVA and VARV. Primary amino acid sequences from VACV WR C16 (W) protein and its orthologues in VACV MVA (M) and VARV GBR46 (V) were aligned using ClustalW software. The underscored region (amino acids 157–331) represents the C-terminal portion used in Figure 2C. (TIF) [file ppat.1003649.s001.tif]

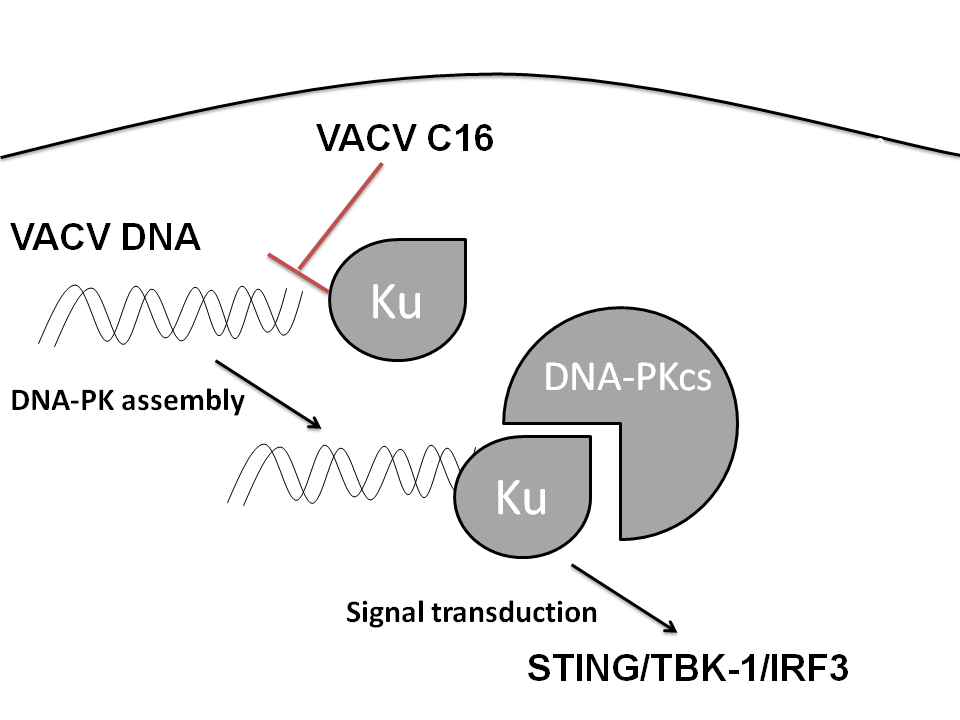

Supplement: Figure S2 — Proposed model of C16 action. C16 inhibits the binding of Ku to DNA thereby preventing the assembly of DNA-PK on VACV DNA and subsequent signal transduction via IRF3. (TIF) [file ppat.1003649.s002.tif]

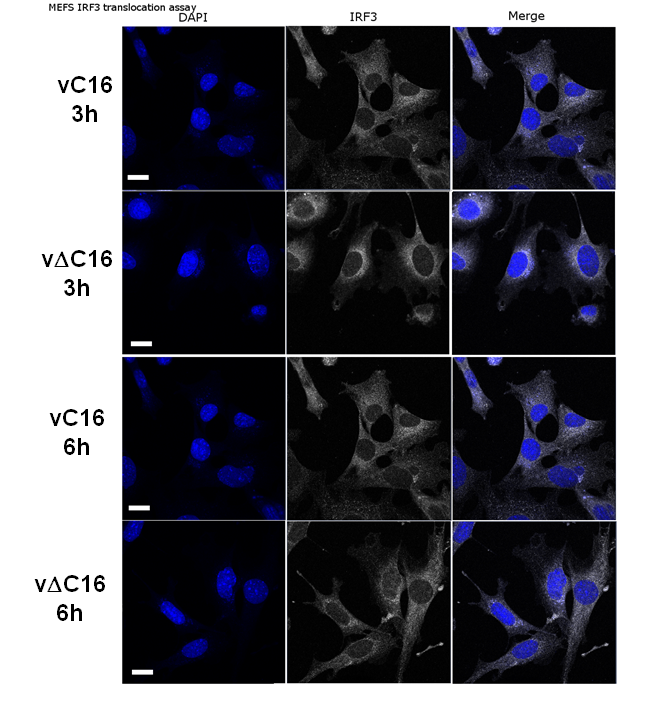

Supplement: Figure S3 — WR infection does not lead to IRF-3 activation. MEFs were infected with either wild-type WR VACV (vC16) or a recombinant virus lacking C16 (vΔC16) for 3 or 6 hours as indicated and stained for IRF-3. Scale bar; 10 µm. (TIF) [file ppat.1003649.s003.tif]
